# Anterior Maxillary Odontogenic Keratocyst Mimicking an Infected Radicular Cyst: A Case Report

**DOI:** 10.7759/cureus.117999

**Published:** 2026-10-16

**Authors:** Laavanya Marimuthu, Sri Devi, A Saneem Ahamed, N S Rajessh

**Affiliations:** 1 Department of Oral and Maxillofacial Surgery, Thai Moogambigai Dental College and Hospital, Chennai, IND; 2 Department of Oral and Maxillofacial Surgery, Priyadarshini Dental College and Hospital, Thiruvallur, IND

**Keywords:** 5-fluorouracil, case report, curettage, enucleation, marsupialization, mixed variant, odontogenic keratocyst, orthokeratinized, parakeratinized, radicular cyst

## Abstract

Odontogenic keratocyst (OKC) is a developmental odontogenic cyst known for its aggressive behavior, characteristic histology, and high recurrence rate. It is typically lined by parakeratinized epithelium, while a purely orthokeratinized lining is now recognized as a separate entity, the orthokeratinized odontogenic cyst (OOC). Lesions showing both types of keratinization are rare and can pose diagnostic and management challenges.

We report a 38-year-old man with no relevant medical history who presented with a six-month history of continuous, dull pain in the upper left anterior region, aggravated by mastication and radiating to the head. Clinical examination showed tenderness on percussion in teeth 21 to 25. An orthopantomogram (OPG) revealed a well-defined cystic radiolucency extending from 21 to 25. Aspiration cytology suggested an infected radicular cyst. After endodontic treatment of the involved teeth, the lesion was enucleated with apicectomy of 21, 22, and 23. Histopathology of the specimen revealed a cyst lined by keratinized stratified epithelium five to six cells thick, with a palisaded, hyperchromatic basal layer and daughter cysts in the connective tissue wall, as well as both orthokeratinized and parakeratinized areas, consistent with a mixed variant of OKC. Subsequently, the cavity was reopened, topical 5-fluorouracil (5-FU) was applied, and periodic iodoform-paraffin gauze packing was performed. The patient remains under follow-up.

This case underlines that OKC can mimic inflammatory cysts, that final histopathology is essential, and that the mixed variant needs careful distinction from the OOC because their behavior and management differ.

## Introduction

Odontogenic keratocyst (OKC) was first described by Philipsen in 1956 and accounts for a substantial share of all jaw cysts. Its classification has changed over time. The 2005 WHO classification renamed it "keratocystic odontogenic tumor" because of its neoplastic-like behavior, but the 2017 WHO classification returned it to the cyst category, and the 5th edition of the WHO Classification of Head and Neck Tumours (2023) retains this [1,2]. The WHO defines OKC as a developmental odontogenic cyst characterized histologically by a thin, parakeratinized stratified squamous epithelial lining with palisaded, hyperchromatic basal cells. It tends to grow along the marrow spaces with little bone expansion and has a notable recurrence rate [3,4].

The orthokeratinized odontogenic cyst (OOC) is defined as a developmental cyst lined by orthokeratinized stratified squamous epithelium, typically with a granular cell layer and a flattened, non-palisaded basal layer. OOC behaves less aggressively than OKC and rarely recurs [5]. Occasional lesions show both parakeratinized and orthokeratinized areas within the same lining. The fifth edition has no separate "mixed" category, and lesions with both types of keratinization in the same lining are classified by the WHO criteria for OKC or OOC. Such lesions are rarely reported and poorly characterized.

Odontogenic cysts of the anterior maxilla are also often mistaken clinically and radiographically for radicular cysts, particularly when adjacent teeth are non-vital or tender. This case is reported because of its rare histology showing both ortho- and parakeratinized epithelium, its initial mimicry of an infected radicular cyst, and its management.

## Case presentation

A 38-year-old man reported to the Department of Oral and Maxillofacial Surgery with a six-month history of pain and discoloration in the upper left front tooth region. The pain was gradual in onset, continuous, aggravated on mastication, relieved by medication, and radiating to the head. He also gave a history of trauma to the upper front tooth 10 years ago. He had no relevant medical, family, or drug history.

On intraoral examination, diffuse swelling measuring 3 × 1.5 cm was observed in the left anterior maxillary region, extending from the midline posteriorly to the anterior wall of the maxillary sinus. The swelling was firm in consistency, non-tender, and had well-defined margins. Teeth 21-25 were tender on percussion, with an Ellis class I fracture in relation to tooth 21. Pulp vitality testing was performed, and tooth 21 was found to be non-vital. A sinus tract opening with pus discharge was observed in the periapical region of tooth 21.

An orthopantomogram (OPG) showed a well-defined radiolucency with a corticated border extending from the region of teeth 21-25 anteroposteriorly and from the nasal floor to the apical third of the roots of teeth 21-25 superoinferiorly (Figure 1).

**Figure 1:**
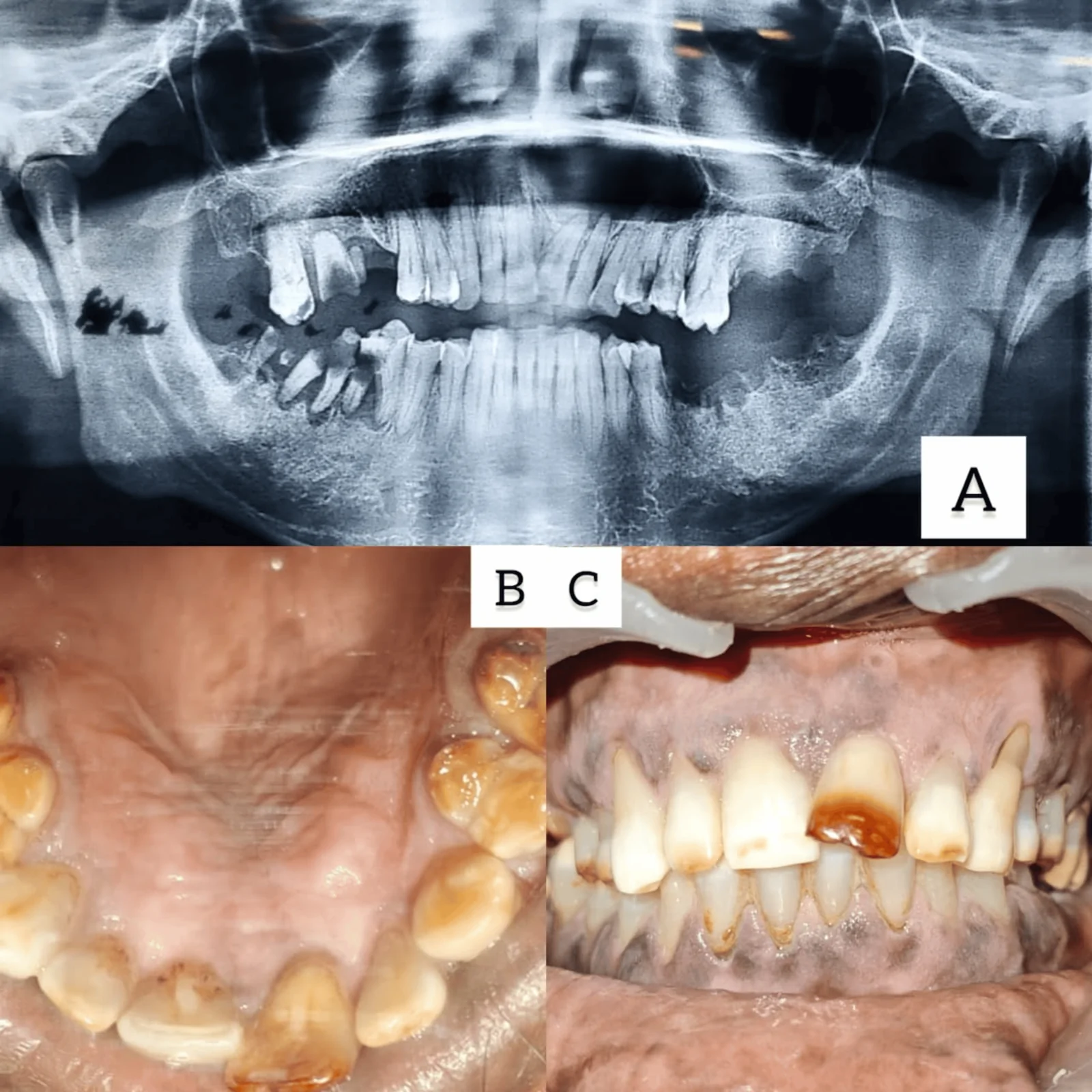
A) Preoperative OPG reveals a well-defined radiolucent lesion involving the roots of 21-25. B) Palatal swelling evident in relation to 21-24. C) Discoloration of 21. OPG: orthopantomogram

Fine-needle aspiration was performed, and the fluid was blood-tinged in nature and cytological examination showed infected pus cells, leading to a provisional diagnosis of an infected radicular cyst. Endodontic treatment of 21-25 was completed before surgery, and enucleation under local anesthesia was planned.

Surgical procedure

Infraorbital, nasopalatine, greater palatine, and posterior superior alveolar nerve blocks were administered. A sulcular incision was placed from 11 to 25, with a vertical releasing incision between 11 and 12 using a No. 15 blade, and a mucoperiosteal flap was elevated. The cystic cavity was accessed with a No. 8 round bur. The lesion was enucleated, with the content noted to be cheesy material, and apicectomy of 21, 22, and 23 was performed. The cavity was irrigated thoroughly with povidone-iodine solution and closed with 3-0 silk sutures. The specimen was sent for histopathological examination (Figure 2).

**Figure 2:**
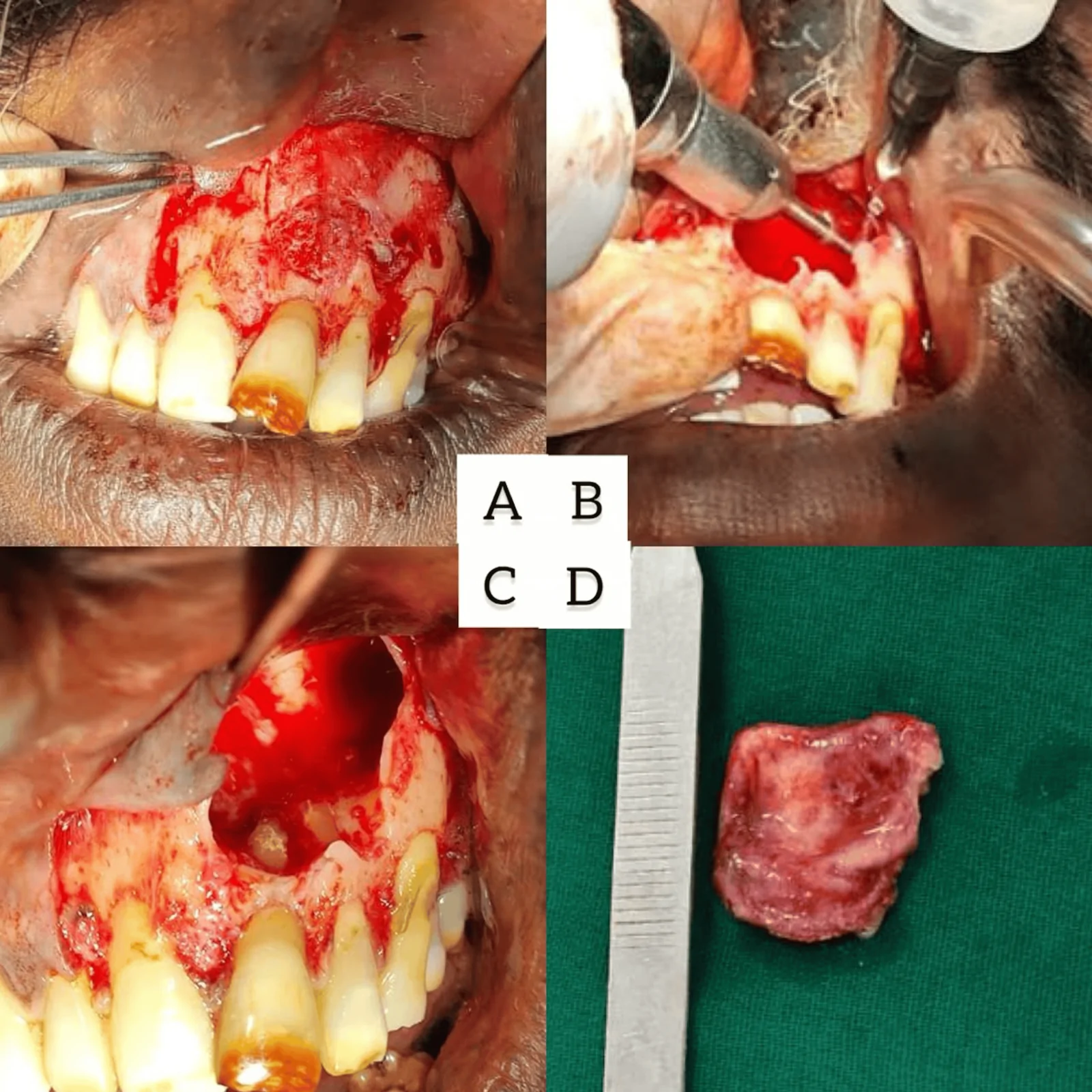
A) Full-thickness mucoperiosteal flap elevated to reveal the cystic opening; B) deroofing of the cystic cavity performed with a No. 8 round bur; C) cyst enucleation and apicectomy performed in relation to 21, 22, and 23; D) specimen measuring 3 × 2 cm sent for histopathological examination.

Histopathology

The cystic lining was keratinized stratified squamous epithelium five to six cell layers thick. The surface showed both orthokeratinized and parakeratinized areas, with orthokeratinization predominating in most areas. The basal layer was palisaded with hyperchromatic nuclei. The fibrous connective tissue wall showed numerous chronic inflammatory cells and two daughter cysts. These findings were consistent with orthokeratinized OKC (Figure 3).

**Figure 3:**
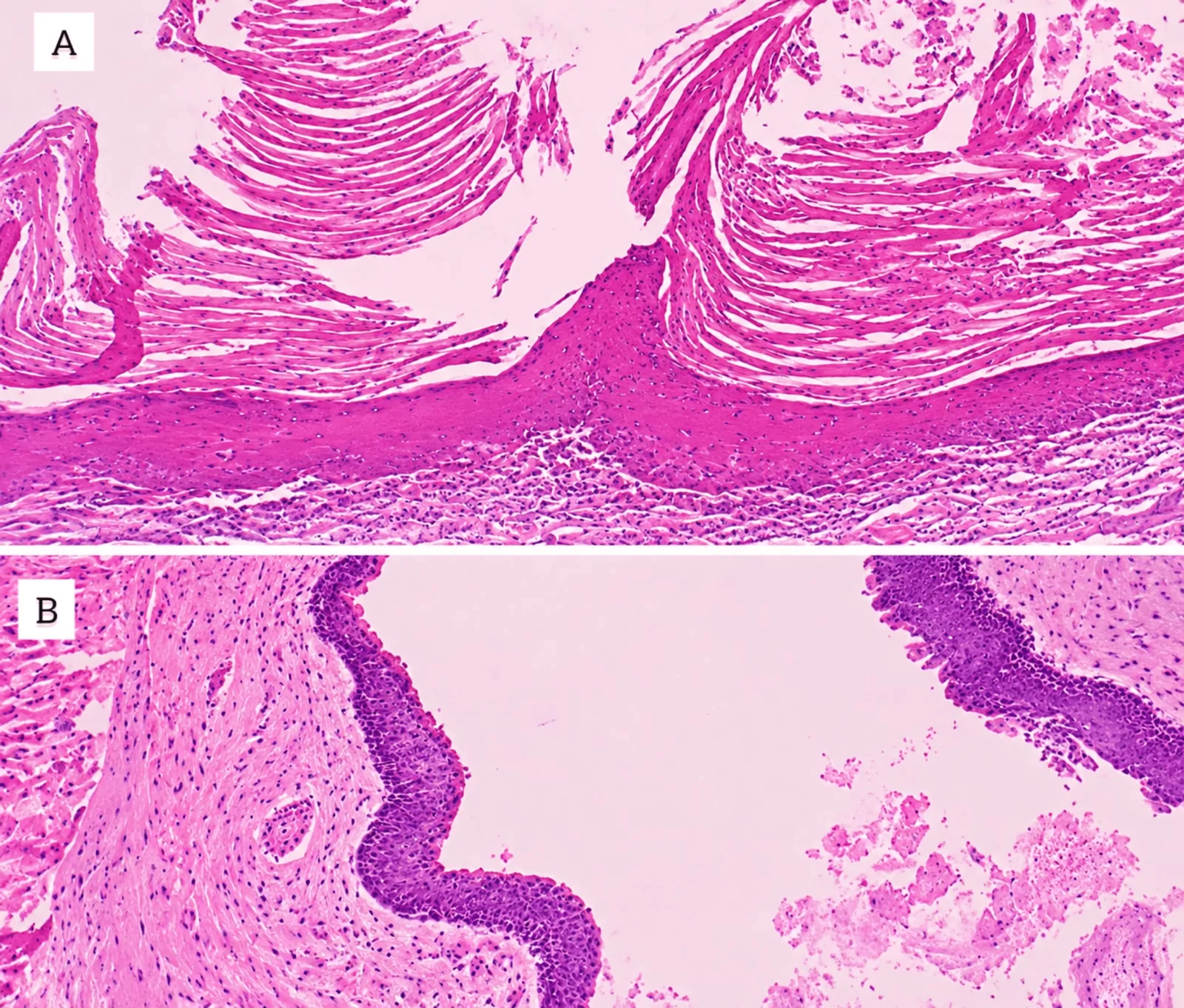
Histopathological examination demonstrating the keratinization pattern of the cystic lining. A) Parakeratinized stratified squamous epithelial lining showing a corrugated keratin surface and palisaded basal cells; B) Orthokeratinized epithelial lining showing a prominent keratin layer and underlying granular cell layer (H&E stain; 10× magnification).

Further management

In view of the unexpected histopathological diagnosis of OKC, the patient was recalled after one week, and the previously operated site was reopened for reassessment. On inspection, the cavity was found to contain granulation tissue, with no identifiable residual cystic lining. The cavity was thoroughly inspected and maintained as an open cavity for continued local management and secondary healing. 5-Fluorouracil (5-FU) cream-soaked roller gauze was placed within the cavity and removed after 24 hours. Subsequently, the cavity was periodically packed with iodoform- and paraffin-soaked roller gauze at 15-day intervals to maintain cavity patency and facilitate healing. The patient showed a significant reduction in pain and swelling during follow-up. At the time of reporting, three months of clinical and radiographic follow-up were available, and the patient remained under continued clinical and radiographic follow-up (Figure 4).

**Figure 4:**
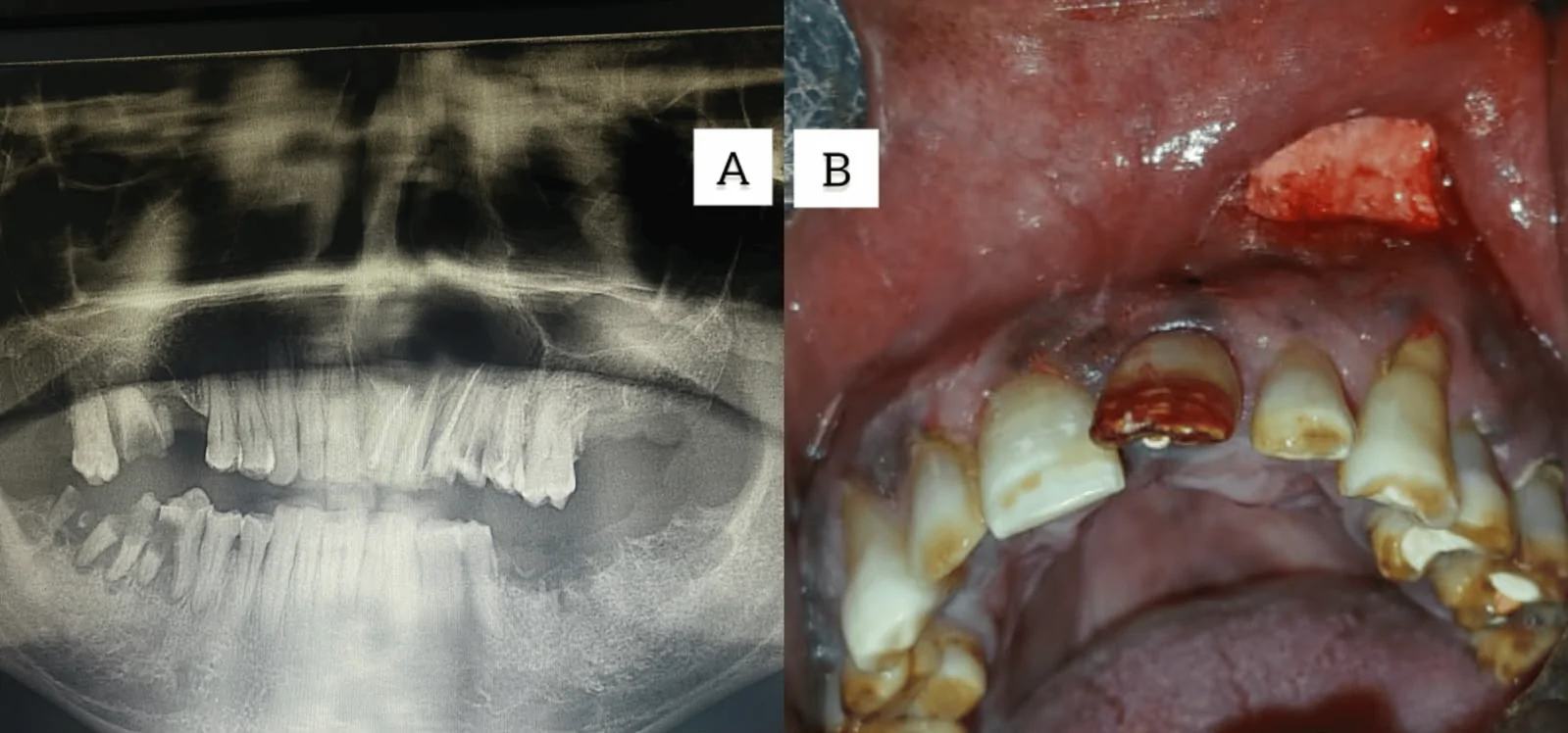
A) Postoperative OPG reveals the cystic cavity at the 3-month follow-up. B) Periodic packing of the cystic cavity with iodoform-soaked ribbon gauze was performed every 15 days. OPG: orthopantomogram

## Discussion

OKC represents a significant share of odontogenic cysts and most often occurs in the mandibular posterior region and ramus, with a peak in the second to fourth decades [4]. Anterior maxillary lesions like the present one are less typical and, as here, may mimic periapical inflammatory lesions. Contributing factors include tenderness on percussion, secondary infection, and the involvement of multiple teeth. Pulp vitality testing, aspirate analysis, and radiographic assessment before endodontic treatment could help avoid a misdiagnosis. Aspirate findings alone can mislead since secondary infection can mask the keratinous content that suggests OKC.

The main differential diagnoses for a well-defined anterior maxillary radiolucency involving several teeth are as follows. A radicular cyst is associated with a non-vital tooth, usually shows a corticated radiolucency at the root apex with loss of the lamina dura, and is histologically lined by non-keratinized stratified squamous epithelium with prominent inflammation and no palisaded basal layer [5]. A dentigerous cyst surrounds the crown of an unerupted tooth at the cementoenamel junction and has a thin, non-keratinized lining [5]. A lateral periodontal cyst is a small, well-circumscribed radiolucency on the lateral surface of a vital tooth root, lined by thin, non-keratinized epithelium with focal plaque-like thickenings [5,6]. A nasopalatine duct cyst lies in the anterior midline of the palate, between and behind the central incisors, and is often heart-shaped on radiographs. It is lined by variable epithelium, including respiratory type, with neurovascular bundles in the wall [5]. A unicystic ameloblastoma can appear as a unilocular radiolucency with root resorption and shows a basal layer of palisaded, reverse-polarized cells with subnuclear vacuolization [5]. A glandular odontogenic cyst shows a multilocular radiolucency with mucous cells, cilia, and microcystic spaces in a non-keratinized lining [6]. In our patient, the keratinized, corrugated lining with a palisaded, hyperchromatic basal layer and daughter cysts allowed the other entities to be excluded histologically.

The OOC was first described by Wright in 1981, who showed that it differs from the parakeratinized OKC in behavior, with fewer recurrences and a rarer association with nevoid basal cell carcinoma syndrome [5]. The 5th edition of the WHO Classification of Head and Neck Tumours (2023) treats it as a distinct entity, with a granular layer, a flat basal layer without palisading, and none of the PTCH1 mutations typical of OKC [5]. The WHO definitions rest on the epithelial lining: OKC is characterized by a thin, parakeratinized lining with palisaded, hyperchromatic basal cells, while OOC is lined by orthokeratinized epithelium.

Our lesion showed areas of both parakeratinization and orthokeratinization, with orthokeratin predominating in most areas. It also had a palisaded, hyperchromatic basal layer, a five- to six-cell-thick lining, and daughter cysts. Because these features meet the WHO histological criteria for OKC, the lesion was diagnosed as OKC, with foci of orthokeratinization. Assigning such a lesion correctly is important because the two entities are managed differently. OOC is generally managed conservatively since enucleation alone is usually sufficient, recurrence is uncommon, and routine follow-up is adequate [5]. OKC, in contrast, recurs more often because of its thin, friable lining, daughter cysts, and growth along the marrow spaces, so simple enucleation is often not enough. It is therefore treated with enucleation plus adjuncts such as Carnoy's solution or peripheral ostectomy, or with decompression/marsupialization followed by enucleation, and it needs longer radiographic surveillance and clinical screening for nevoid basal cell carcinoma syndrome [3,4,6]. Sampling error is another concern: an incisional biopsy might have sampled only the orthokeratinized areas or only the inflamed lining, leading to misclassification. This supports examination of the whole enucleated specimen and adequate sectioning.

Daughter (satellite) cysts in the wall are considered a factor in recurrence after enucleation because remnants can be left behind. Inflammation, as seen here, can alter the epithelium and obscure the classic features, which may partly explain why the initial impression was an infected radicular cyst [4].

Recurrence rates of OKC vary by modality, and simple enucleation is generally reported to have higher recurrence than approaches with adjuncts or resection, unlike OOC [4,7]. Marsupialization or decompression can reduce cyst size and thicken the lining and has been proposed as a definitive treatment in selected cases [6]. Topical 5-FU has been proposed as a targeted adjunct based on its antimetabolite action on the rapidly proliferating epithelium [8]. In our patient, 5-FU application followed by periodic packing was chosen due to the proximity to the maxillary sinus and nasal floor and preservation of adjacent teeth. Because recurrence can occur many years later, long-term follow-up with periodic clinical and radiographic review is required [3,4].

Limitations

This is a single case with limited follow-up of only three months. No molecular testing was done. Only low-power photomicrographs were available.

## Conclusions

OKC of the anterior maxilla can mimic an infected radicular cyst, and histopathology of the whole specimen is essential for a definitive diagnosis. The orthokeratinized OKC is rare, and features such as basal palisading, hyperchromatic nuclei, and daughter cysts help separate it from the OKC. Clinicians should treat such lesions as OKCs, use appropriate adjunctive measures, and maintain long-term follow-up because of the risk of recurrence.
